# A natural language processing algorithm accurately classifies steatotic liver disease pathology to estimate the risk of cirrhosis

**DOI:** 10.1097/HC9.0000000000000403

**Published:** 2024-03-29

**Authors:** Marc S. Sherman, Prasanna K. Challa, Eric M. Przybyszewski, Robert M. Wilechansky, Eugenia N. Uche-Anya, Ashley T. Ott, Jessica McGoldrick, Wolfram Goessling, Hamed Khalili, Tracey G. Simon

**Affiliations:** 1 Division of Gastroenterology, Department of Medicine, Massachusetts General Hospital, Harvard Medical School, Boston, MA, USA; 2 Brigham Division of Genetics, Department of Medicine, Brigham and Women's Hospital, Harvard Medical School, Boston, MA, USA; 3 Division of Gastroenterology and Hepatology, Department of Medicine, Stanford University, Stanford, California, USA

## Abstract

**Background:**

Histopathology remains the gold standard for diagnosing and staging metabolic dysfunction–associated steatotic liver disease (MASLD). The feasibility of studying MASLD progression in electronic medical records based on histological features is limited by the free-text nature of pathology reports. Here we introduce a natural language processing (NLP) algorithm to automatically score MASLD histology features.

**Methods:**

From the Mass General Brigham health care system electronic medical record, we identified all patients (1987–2021) with steatosis on index liver biopsy after excluding excess alcohol use and other etiologies of liver disease. An NLP algorithm was constructed in Python to detect steatosis, lobular inflammation, ballooning, and fibrosis stage from pathology free-text and manually validated in >1200 pathology reports. Patients were followed from the index biopsy to incident decompensated liver disease accounting for covariates.

**Results:**

The NLP algorithm demonstrated positive and negative predictive values from 93.5% to 100% for all histologic concepts. Among 3134 patients with biopsy-confirmed MASLD followed for 20,604 person-years, rates of the composite endpoint increased monotonically with worsening index fibrosis stage (*p* for linear trend <0.005). Compared to simple steatosis (incidence rate, 15.06/1000 person-years), the multivariable-adjusted HRs for cirrhosis were 1.04 (0.72–1.5) for metabolic dysfunction–associated steatohepatitis (MASH)/F0, 1.19 (0.92–1.54) for MASH/F1, 1.89 (1.41–2.52) for MASH/F2, and 4.21 (3.26–5.43) for MASH/F3.

**Conclusions:**

The NLP algorithm accurately scores histological features of MASLD from pathology free-text. This algorithm enabled the construction of a large and high-quality MASLD cohort across a multihospital health care system and disclosed an accelerating risk for cirrhosis based on the index MASLD fibrosis stage.

## Graphical abstract

**Figure:**
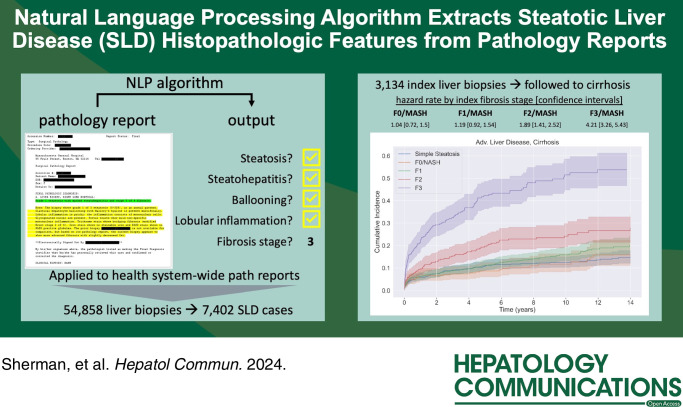

## INTRODUCTION

Metabolic dysfunction–associated steatotic liver disease (MASLD) and its progressive counterpart metabolic dysfunction–associated steatohepatitis (MASH) represent the fastest-growing clinical burden in hepatology.^1^^,^^2^ Large cohorts are needed to accurately quantify disease incidence, estimate rates of disease progression, and identify risk factors.^3^^,^^4^ Assembling such cohorts is increasingly tenable due to the wide availability of large electronic medical record databases.^5^ However, studies employing such data sets tend to estimate MASLD diagnoses using noninvasive, non-narrative–based diagnostics, ignoring radiology and liver pathology because the free-text cannot be processed accurately in an automated manner. However, an appropriate diagnosis of MASLD requires either imaging or histopathology, with the latter representing the gold standard for both diagnosis and staging disease severity.^6^ The omission of such diagnostic data fundamentally limits the granularity and specificity of conclusions that one may draw from large cohort studies.

An automated approach to analyzing the free text describing liver biopsy results represents an appealing and powerful potential tool for increasing the fidelity and scope of electronic medical record-based cohorts of MASLD. While such data are often available at the center-level, and in some small nations,^5^ their interpretation and collation for cohort construction pose significant challenges. Not all features of MASLD or MASH are explicitly noted in every document, and there is wide interperson and intraperson stylistic variability in the reporting of each histologic feature.^7^ Pathologists may emphasize the absence of features, or list differential diagnoses that ought to be considered or excluded. Furthermore, the currently accepted scoring rubric for MASH was established in 2005,^8^ resulting in evolution over time in descriptions of key histopathologic features. These challenges make simple free-text matching and if-then logic inadequate for high-quality automated data extraction.

Natural language processing (NLP) is a rapidly maturing discipline of machine learning that aims to interpret human prose and offers a powerful potential solution to the interpretation of pathology free-text. Early efforts demonstrated that NLP was capable of automatically scoring tumor histology grade and stage^9^^–^^11^ or morphology^12^ from surgical pathology reports for solid tumors. While accurate, these successes required niche computational linguistic expertise in sentence parsing, parts of speech labeling, feature identification, and context-specific training,^10^^,^^12^ thus limiting their broad application. The recent development of more generalized NLP algorithms pre-trained on medical text which incorporate these prerequisite programmatic routines^13^^–^^16^ offers an opportunity to understand medical documentation without having to train a new algorithm for each new text corpus, and has been applied to annotate radiology,^17^ progress,^15^^,^^16^^,^^18^^,^^19^ and colonic anatomical pathology reports.^16^

In MASLD, much of the NLP-directed efforts to date have focused on detecting MASLD from narrative clinical documentation like progress notes.^18^^,^^20^ However, none have developed and validated an NLP algorithm to ascertain and stage MASLD from liver histology free-text. Here we asked how a pre-trained NLP algorithm could be applied to interpreting a liver biopsy report according to MASH Clinical Research Network diagnostic criteria for MASLD and fibrosis staging.^8^ We validate this tool and use it to construct a large, system-wide cohort of patients with biopsy-proven MASLD and estimate rates of progression to advanced liver disease and HCC.

## METHODS

### Study population and histopathology data set

**FIGURE 1:**
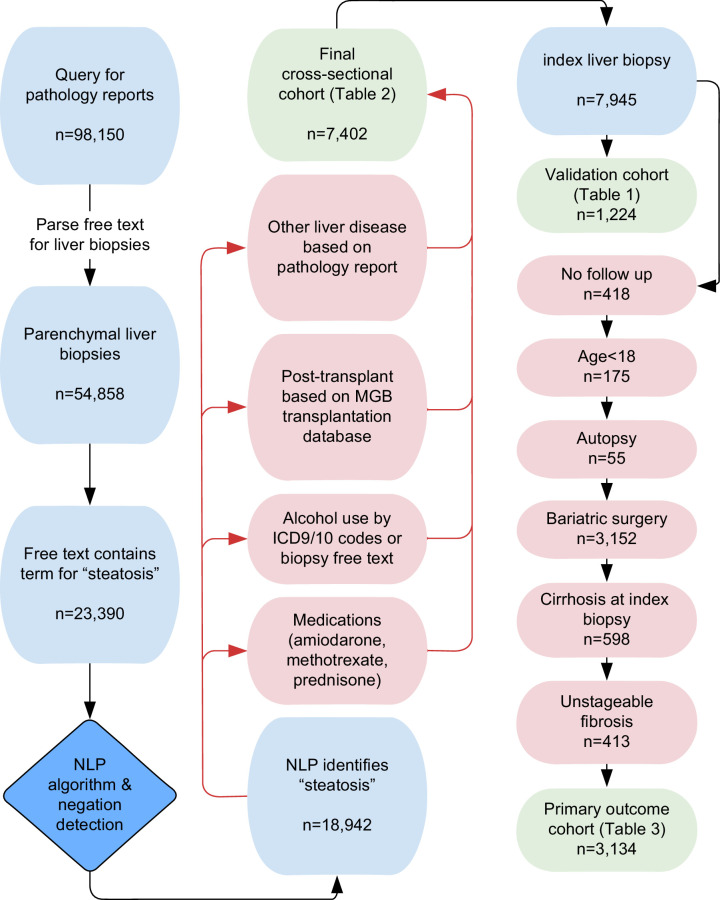
Flow diagram for algorithm and cohort construction. MGB-system–wide pathology reports were requested and screened for parenchymal liver biopsies. After excluding other liver diseases and validating the NLP algorithm, a cross-sectional cohort was created capturing patient characteristics at the time of liver biopsy, and a second cohort was followed from index biopsy to incident cirrhosis or HCC. Abbreviations: ICD, International Classification of Diseases; MGB, Mass General Brigham; NLP, natural language processing.

The study population includes patients seen at a Mass General Brigham (MGB) a Boston-based regional health care system that includes Massachusetts General, Brigham and Women’s, Newton-Wellesley, Brigham and Women’s Faulkner, Cooley-Dickinson, Salem, Spaulding, Martha’s Vineyard, Nantucket Cottage, Wentworth-Douglass hospitals, and associated community health centers. The MGB includes tertiary-care academic medical centers, satellite teaching hospitals, and community hospitals which are primarily urban and suburban. Patient data were obtained from the Research Patient Database Registry (RPDR), a system-wide repository for all retrospective electronic health care records from the MGB system. This study was approved by the MGB Institutional Review Board. The RPDR was queried on March 22, 2021 for all patients between the years 1987 and 2021 with a pathology report from one of the MGB hospitals containing the term “hepatic” or “liver.” From each histopathology report, headers and footers containing demographic information, clinical context, and gross specimen details were parsed for removal using custom Python code, leaving only the diagnostic text corresponding to findings and impressions or conclusions. These reports were further filtered using simple text matching criteria to identify bona fide liver biopsies, by excluding reports describing biopsies of other organs that also mentioned “liver” or “hepatic,” as well as liver fine-needle aspirations and biopsies of hepatic tumors (Figure 1).

### NLP algorithm

The NLP algorithm was created in Python drawing from spacy^14^^,^^21^ and negex/negspacy^22^^,^^23^ NLP packages, and using the United Medical Language System corpus.^13^ Specifically, the Allen AI scispacy^24^ en_core_sci_lg package pre-trained on medical text was used. The algorithm parses the diagnostic component of a pathology report and returns paired outputs containing (1) a medical concept, and (2) the presence or absence of that feature. Importantly, the concepts were selected automatically from the curated unified medical language system medical dictionary,^13^ and not manually ascribed, rendering the output an unbiased representation of the pathologist’s assessment. To interpret this raw output, keywords and text search were used to identify key MASLD concepts among the medical concepts (output 1), and to identify biopsies where MASLD was absent or coincident with a second disease. The algorithm code and instructions are available at https://github.com/prasanna324/RPDR_cohort_manuscript.

### Definitions of MASLD for NLP algorithm and manual validation

MASLD is diagnosed by the presence of specific histopathologic features. Steatotic liver disease or simple steatosis is defined by the presence of steatosis, or lipid droplet accumulation in the liver. MASH, the progressive form of the disease, is defined by a pattern of liver injury superimposed on steatosis which includes hepatocyte ballooning and lobular inflammation.^6^^,^^8^^,^^25^ The MASH clinical research network (CRN) further established a staging system for fibrosis for which pericellular fibrosis in zone 3 is defined as fibrosis stage 1 (F1), pericellular fibrosis in zone 3 and fibrosis in zone 1 is defined as stage 2 (F2), bridging fibrosis is defined as stage 3 (F3), and nodule formation is defined as stage 4 (F4) and equivalent to cirrhosis.^8^

To adapt MASLD feature definitions and staging to our data set, a scoring rubric was developed by lead authors (Marc S. Sherman and Tracey G. Simon) to guide both the algorithm and validators. Four core MASLD terms were defined as present or absent: steatosis, lobular inflammation, ballooning, and MASH/steatohepatitis (as a summative term). For these terms and their synonyms, any assertion of the pathologist of any of these concepts’ presence or absence was scored as such, while omissions were recorded as absent. Descriptive diminutives like “minimal,” “patchy,” and “borderline” were scored as present. Portal inflammation and synonyms were not considered to reflect lobular inflammation, whereas lobular or zone 3 inflammation was scored as positive for lobular inflammation. For all cohort and outcomes analyses, MASH was defined by a pathologist’s assertion of MASH being present as detected by the NLP algorithm. In the absence of a summative assertion by the reporting pathologist as to the presence of steatohepatitis, MASH was considered positive if all 3 elements of MASH (steatosis, lobular inflammation, and ballooning) were present.^26^

Fibrosis was scored primarily according to the MASH CRN fibrosis system.^8^^,^^25^ The algorithm first identified explicit pathologist assignment of fibrosis stage from the report text, and if absent sought to stage based on the presence of component parts and their synonyms (eg, if no explicit fibrosis stage stated, the presence of perisinusoidal and periportal fibrosis would be scored as F2). As per Brunt and Tiniakos,^27^ we took the highest fibrosis stage present, even if the pathologist indicated a range (eg, “F1-F2” becomes F2, and “F2 with focal areas of bridging” becomes F3). For reported outcomes, fibrosis staging according to MASH CRN criteria superseded the explicit requirement of MASH grading features. For example, a biopsy showing steatosis and lobular inflammation with MASH CRN–defined F2 fibrosis staging was labeled as MASH/F2, despite the absence of ballooning.

### Structured validation of the NLP algorithm

Validation was structured in 2 phases, both as double-blinded manual validation for the 4 MASLD concepts plus the 5 fibrosis stages. In phase 1, biopsies were validated from Massachusetts General Hospital, which comprised over 80% of reports. At least 100 positive examples of each of the 9 concepts were generated randomly, balanced by the decade 1990–2021. Five expert clinicians, blinded to the NLP algorithm interpretation, manually scored a total of 1224 reports. In parallel, another author (Prasanna K. Challa), blinded to the validation results, produced and collated the NLP output for the same reports. One coding error that led to misclassification of cirrhosis was identified after this first round of manual validation (affecting <1% of cases). This was corrected, and the final algorithm was deployed in the second phase, where again at least 100 examples of each of the 9 concepts were generated from liver biopsy reports from 4 additional hospitals within the health care system which have independent pathology departments: Brigham and Women’s Hospital, Faulkner Hospital, Newton-Wellesley Hospital, and North Shore Medical Center.

### Analysis of algorithmic performance and algorithm-clinician disagreement

The results from the manual interpretation and NLP interpretation for each report in the validation cohort were compared, with the calculation of performance metrics, including sensitivity, specificity, negative predictive value, positive predictive value, F1 scores, and support. F1 scores are a machine-learning statistic for overall algorithm fidelity and represent the harmonic mean of precision and recall.^10^ Support grades the adequacy of concept coverage in the validation cohort and is equivalent to the count of true positives for each concept. A structured secondary analysis with blinded rescoring by an additional study member (Marc S. Sherman) was then conducted to identify algorithm and validator error rates, in instances of algorithm-validator disagreement. A qualitative assessment was also conducted to understand patterns in algorithm failure.

### MASLD cohort construction, exclusions, and comorbidities

Once validated, the NLP algorithm was applied to all available liver biopsies within the MGB system to construct a comprehensive, system-wide cohort with biopsy-confirmed MASLD between 1987 and 2021. The cohort inclusion and exclusion criteria are summarized in Figure 1, with detailed definitions in the Supplemental Materials, http://links.lww.com/HC9/A837. Briefly, among all NLP-identified simple steatosis or MASH biopsies, we excluded the presence of other liver diseases, alcohol use, exposure to steatogenic medications, history of bariatric surgery, and posttransplantation status. Matched data from our original histopathology query were used to obtain patient demographics, comorbidities, and outcomes indexed to 12 months before 6 months after the initial diagnosis. Comorbidities examined included smoking, diabetes, hypertension, body mass index (BMI), and hyperlipidemia. Covariates included aspirin therapy and statin therapy (Supplemental Materials, http://links.lww.com/HC9/A837).

### Definitions of outcomes

The primary outcome was a diagnosis of advanced liver disease, a composite including incident cirrhosis or a liver decompensation event, including ascites, spontaneous bacterial peritonitis, bleeding esophageal or gastric varices, or HE, using validated International Classification of Diseases (ICD) algorithms with established PPVs (Supplemental Materials, http://links.lww.com/HC9/A837).^28^ In a secondary, prespecified analysis we also ascertained incident HCC using analogous, validated ICD algorithms (Supplemental Materials, http://links.lww.com/HC9/A837).

### Outcomes statistical analysis

Index time was considered as the date of the first liver biopsy meeting criteria for MASLD without cirrhosis, and the endpoint was documented as the first occurrence of advanced liver disease as defined above. Cumulative incidence curves were used to calculate incidence rates bounded by 95% CIs and results were reported in events per 1000 patient years. Cox proportional hazard models were used to estimate multivariable-corrected hazard rates which accounted for age at the index date, sex, BMI, diabetes, dyslipidemia, smoking status, aspirin therapy, and statin therapy. Data were prepared in Python as above, and then statistical analysis was conducted in R using previously published scripts.^5^

Multiple sensitivity analyses were conducted to ascertain the robustness of the data. Cirrhosis and decompensated events were considered separately. Each outcome was examined excluding events occurring within 30 days after the index biopsy. We performed a sensitivity analysis excluding patients with missing BMI to assess whether BMI missingness affects the multivariate corrected hazard rates. Finally, we examined whether including what would be classified as borderline MASH^26^ as MASH cases affected our risk estimates.

## RESULTS

### Construction and validation of an NLP algorithm

Our query for patients with liver biopsy reports from the RPDR database resulted in 95,150 free-text pathology reports, of which 54,858 were determined to be parenchymal liver biopsies. During the construction of the NLP algorithm and finalizing of the text search terms for post-NLP output filtering, the algorithm appeared to be highly effective at positively identifying cases of steatosis. We therefore used the NLP algorithm to enrich cases where steatosis was present (Figure 1) for validation purposes, and set aside 200 random cases for manual validation of the algorithm’s negative predictive value for the “steatosis” term. Thus, the initial filter for cohort construction and validation contained only cases where the NLP algorithm already detected reports positive for “steatosis” (Figure 1). Subsequent filtering for alcohol use, posttransplant, and other liver diseases, and excluding history of amiodarone, prednisone, and methotrexate resulted in a cohort of 7402 patients, of which 7086 were index biopsies. The validation cohort selected from among this group resulted in 517 biopsy reports from Massachusetts General Hospital (phase 1 validation), 409 reports from Brigham and Women’s Hospital, and 98 reports from Newton-Wellesley Hospital, Wentworth Douglas Hospital, and North Shore Hospital (phase 2 validation).

Agreement was high between the NLP algorithm and manual validators for all categories (Table 1). Positive and negative predictive values ranged from 93.5% to 100%, and >97% for 7 of 9 concepts, respectively. Of the 4 histological features, ballooning (positive predictive value (PPV), 100% and negative predictive value (NPV), 98.5%) and steatosis (PPV, 100% and NPV, 97.5%) showed the best agreement with validators, while lobular inflammation was the poorest (PPV, 99% and NPV, 95%), but still excellent. Among fibrosis stages, only F2 showed slight disagreement (PPV, 93.5% and NPV, 99.4%), while the remainder of stages including an absence of fibrosis (F0) were accurately ascertained by the NLP algorithm. Although the algorithm was primarily created using Massachusetts General Hospital pathology reports as a reference, no clear failure pattern emerged when examining BWH validation or other hospital validation data separately (Supplemental Tables S1–S3, http://links.lww.com/HC9/A837). Finally, reports drawn from the parenchymal liver biopsy cohort which were negative for NLP-detected steatosis showed extraordinary negative predictive values (Supplemental Table S4, http://links.lww.com/HC9/A837; NPV, 99.5%).

**TABLE 1:** Performance characteristics of NLP algorithm for prediction of MASLD concepts

Concept	n^a^	Sensitivity/recall %	Specificity %	PPV/Precision %	NPV %	F_1_ score^b^	Support^a^
“steatosis”	1224	99.9	98.0	99.6	99.5	99.8	1021
“lobular inflammation”	1024	90.9	99.6	99.0	95.9	94.8	328
“ballooning”	1024	96.4	100.0	100.0	98.5	98.2	304
“NASH”^c^	1024	98.6	97.8	97.1	99.0	97.9	444
“cirrhosis”	1024	98.5	99.4	97.6	99.6	98.0	203
Fibrosis F0	1024	96.0	99.2	97.3	98.9	96.6	223
Fibrosis F1	1024	93.5	99.0	96.2	98.3	94.8	216
Fibrosis F2	1024	97.4	98.4	93.5	99.4	95.4	193
Fibrosis F3	1024	98.4	99.5	97.9	99.6	98.2	191
Fibrosis F4	1024	98.0	99.3	97.0	99.5	97.5	198

a Total n is the number of pathology reports evaluated for an element, whether or not the element was present; Support is the actual number of occurrences of this element in the relevant pathology reports.

b F_1_ is the harmonic mean of precision and recall.

c Historic term used here as all NLP-based terms were based on pre-2023 MASLD naming conventions.

Abbreviations: MASLD, metabolic dysfunction–associated steatotic liver disease; NLP, natural language processing; NPV, negative predictive value; PPV, positive predictive value.

Algorithm-validator disagreement, as arbitrated by a second blinded validator, highlighted algorithmic gaps and quantified algorithm and human validator error rates (Supplemental Table S5, http://links.lww.com/HC9/A837 and Supplemental Table 6, http://links.lww.com/HC9/A837). Cumulatively across all validation sets with algorithm-validator disagreement, the algorithm was correct in 36% of cases, the first validator was correct in 62% of cases, and in 2% of cases all 3 disagreed. The overall apparent algorithmic error rate across all categories was 1.7%, while the overall human error rate across all categories was 1.1%. Errors occurred at a rate of ~4:1 favoring the validators in the first 517 cases, but occurred at a rate of 1:1 in the second 507 cases (Supplemental Table S7, http://links.lww.com/HC9/A837). Most algorithm-validator disagreements originated from the lobular inflammation category (n=49, 28%) and fibrosis staging (n=60, 34%). On secondary validator review, this subjectively seemed to derive from more ways to describe both lobular inflammation and the nature of fibrosis, whereas steatosis and ballooning notation were generally binary and with few synonyms.

### Creation of a large, high-quality MASLD cohort from histopathology free text

We then applied this highly accurate, validated NLP algorithm to construct a cross-sectional MASLD cohort of patients across our entire health care system (Table 2). The algorithm-generated cohort identified a steady rise of aspartate aminotransferase (AST) and alanine aminotransferase (ALT), with ALT greater than AST across the fibrosis stage accompanied by a relative decline with the onset of cirrhosis. Cirrhosis was marked by an uptick in PT-INR, total bilirubin, and a reduction in BMI and hypertension. Dyslipidemia was markedly reduced at the onset of cirrhosis.

**TABLE 2:** Cross-sectional cohort characteristics for all MASLD liver biopsies in the Mass General Brigham system 1995–2021

Characteristic	All MASLD (N=7402)	Simple steatosis (N=2632)	MASH/F0 (N=766)	MASH/F1 (N=2110)	MASH/F2 (N=691)	MASH/F3 (N=523)	Cirrhosis (N=680)
Female, %	4439 (59.97)	1743 (66.22)	451 (58.88)	1206 (57.16)	382 (55.28)	278 (53.15)	379 (55.74)
Age at biopsy, y (SD)	48.63 (14.49)	47.36 (14.23)	44.70 (14.59)	47.59 (14.05)	49.60 (14.63)	52.08 (14.52)	57.56 (12.38)
Race/ethnicity, n (%)							
Asian	207 (2.80)	39 (1.48)	30 (3.92)	83 (3.93)	22 (3.18)	15 (2.87)	18 (2.65)
Black	319 (4.31)	155 (5.89)	34 (4.44)	81 (3.84)	20 (2.89)	15 (2.87)	14 (2.06)
Hispanic	370 (5.00)	129 (4.90)	51 (6.66)	104 (4.93)	33 (4.78)	24 (4.59)	29 (4.26)
White	5622 (75.95)	2024 (76.90)	551 (71.93)	1609 (76.26)	529 (76.56)	406 (77.63)	503 (73.97)
Other	377 (5.09)	112 (4.26)	55 (7.18)	119 (5.64)	42 (6.08)	25 (4.78)	24 (3.53)
Not recorded	507 (6.85)	173 (6.57)	45 (5.87)	114 (5.40)	45 (6.51)	38 (7.27)	92 (13.53)
ALT, IU/L, median (IQR)	62.60 (22.00–69.00)	47.56 (18.00–42.00)	68.71 (27.00–76.00)	68.60 (25.00–75.00)	82.97 (32.00–101.00)	88.94 (37.00–104.00)	55.61 (26.00–64.00)
AST, IU/L, median (IQR)	52.03 (21.00–51.00)	41.84 (19.00–33.00)	44.82 (24.00–46.00)	53.51 (22.00–51.00)	64.24 (29.00–74.00)	81.26 (35.00–87.00)	65.09 (33.00–79.00)
Total bilirubin, mg/dL, median (IQR)	0.77 (0.30–0.60)	0.74 (0.30–0.60)	0.60 (0.30–0.60)	0.64 (0.30–0.60)	0.77 (0.30–0.70)	1.02 (0.40–0.80)	1.43 (0.50–1.20)
PT-INR (SD), median (IQR)	1.08 (1.00–1.10)	1.08 (1.00–1.10)	1.04 (1.00–1.10)	1.06 (1.00–1.10)	1.07 (1.00–1.10)	1.10 (1.00–1.10)	1.23 (1.10–1.30)
BMI, kg/m^2^ (SD)	40.80 (10.82)	42.02 (10.78)	39.86 (10.34)	41.65 (10.95)	39.43 (10.47)	37.07 (10.04)	35.77 (10.06)
Diabetes, %	27.68	21.35	22.19	33.51	30.68	36.52	30.44
Dyslipidemia, %	40.10	39.10	40.73	46.30	40.52	39.58	23.97
Hypertension, %	44.23	43.24	42.69	50.24	43.70	42.83	32.79

Abbreviations: ALT, alanine aminotransferase; AST, aspartate aminotransferase; BMI, body mass index; MASH, metabolic dysfunction–associated steatohepatitis; MASLD, metabolic dysfunction–associated steatotic liver disease.

### Progression to advanced liver disease by MASLD fibrosis stage

After narrowing the cross-sectional cohort (Table 2) to include only index biopsies, excluding patients with inadequate follow-up (Figure 1), and excluding patients with liver biopsies performed at the time of bariatric surgery (n=3152), 3134 patients were included in the risk analysis with baseline characteristics shown in Table 3. Among patients with biopsy-proven MASLD at index liver biopsy, 64.2% had precirrhotic MASH.

**TABLE 3:** Longitudinal cohort characteristics for patients with noncirrhotic MASLD on index liver biopsy

Characteristic	All MASLD (N=3134)	Simple steatosis (N=1122)	MASH/F0 (N=369)	MASH/F1 (N=934)	MASH/F2 (N=382)	MASH/F3 (N=327)
Female, %	53.29	57.4	52.85	48.93	52.09	53.52
Age at the index date, y (SD)	51.07 (13.89)	51.99 (14.27)	46.95 (13.95)	50.36 (13.5)	51.09 (13.77)	54.6 (12.43)
Race/ethnicity, %						
Asian	4.56	2.76	6.23	6.75	3.66	3.67
Black	3.25	3.57	3.79	3.1	2.88	2.45
Hispanic	4.75	3.3	7.32	5.89	4.45	3.98
White	76.23	80.04	68.02	73.88	76.44	78.9
Other	4.66	2.58	6.78	5.03	6.54	4.89
Not recorded	6.54	7.75	7.32	5.25	5.76	6.12
Years of follow-up^a^, median (IQR)	4.3 (9.40)	3.56 (10.88)	5.49 (8.54)	4.97 (9.1)	4.76 (9.42)	2.78 (6.98)
Start of follow-up, %						
1987–1999	13.34	18.89	6.78	11.78	8.9	11.31
2000–2009	31.78	34.22	30.89	30.3	31.15	29.36
2010–2021	54.88	46.88	62.33	57.92	59.95	59.33
Hospital, %						
Massachusetts General Hospital	66.08	55.79	79.95	70.77	69.9	67.89
Brigham and Women’s Hospital	28.53	39.04	16.8	24.2	24.08	23.24
Faulkner Hospital	2.11	1.43	2.17	2.03	2.88	3.67
Newton-Wellesley Hospital	1.75	2.5	0.27	1.39	2.09	1.53
North Shore Medical Center	1.53	1.25	0.81	1.61	1.05	3.67
BMI, %						
<30	21.54	24.96	20.6	21.84	15.71	16.82
≥30	27.35	17.11	36.86	30.62	35.08	33.33
Missing	51.12	57.93	42.55	47.54	49.21	49.85
Diabetes, %	18.83	14.62	12.47	20.34	21.47	33.03
Dyslipidemia, %	28.62	22.73	32.25	31.91	30.1	33.64
Hypertension, %	31.40	27.18	32.25	33.4	32.72	37.61
Smoking status, %						
Never	22.11	18.0	26.29	23.77	26.18	22.02
Ever	14.52	12.92	15.45	15.2	14.4	17.13
Missing	63.37	69.07	58.27	61.03	59.42	60.86
Aspirin therapy, %	14.10	10.96	13.55	14.99	15.97	20.8
Statin therapy, %	22.40	19.52	21.41	23.77	24.87	26.61

a Year of follow-up: Considered any cirrhosis, decompensated liver disease, or HCC.

Abbreviations: BMI, body mass index; MASH, metabolic dysfunction–associated steatohepatitis; MASLD, metabolic dysfunction–associated steatotic liver disease.

**FIGURE 2:**
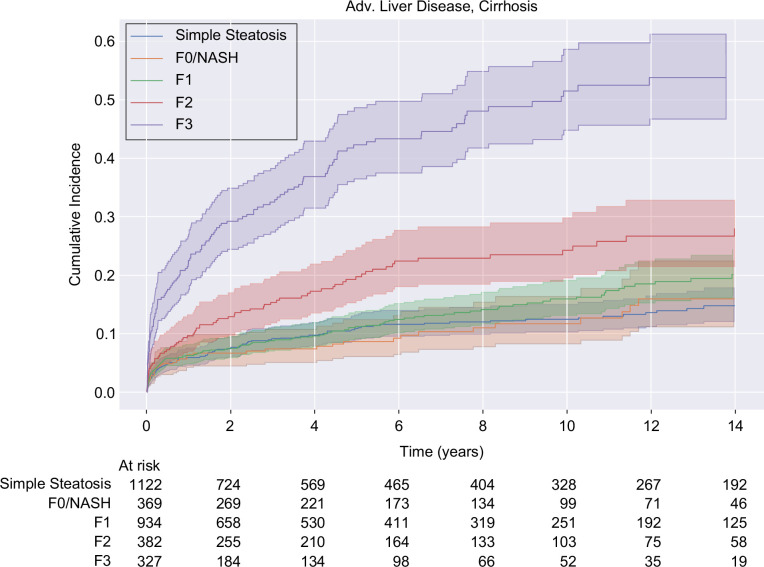
Cumulative incidence of cirrhosis or decompensated liver disease based on fibrosis/MASLD stage at index biopsy. Abbreviation: MASLD, metabolic dysfunction–associated steatotic liver disease.

In our risk analysis, we documented 486 cases of advanced liver disease (20,604 person-years). The risk of advanced liver disease increased from steatosis to MASH/F0, through F3 (trend, *p*<0.005, Table 4). Hazard rates for the development of the primary outcome did not significantly differ from steatosis until stage F2, though F1 trended toward significance (*p*=0.18). The risk of advanced liver disease nearly doubled from F0-F1 to F2, and more than doubled from F2 to F3. The absolute incidence rates were ~20%–30% lower by excluding outcomes occurring within 30 days of index biopsy, without changing the overall pattern; however, hazard rates were not materially changed in this sensitivity analysis (Supplemental Table S7, http://links.lww.com/HC9/A837). In addition, excluding patients with missing BMI did not substantially alter the hazard rates (Supplemental Table S8, http://links.lww.com/HC9/A837). Cumulative incidence curves highlight that outcomes are only modestly different for steatosis through MASH/F1 but clearly stratify with increasing occurrence of the primary outcome for F2 and F3 (Figure 2).

**TABLE 4:** Longitudinal cohort risk analysis for primary endpoint (cirrhosis or decompensated liver disease)

	All MASLD (N=3134)	Simple steatosis (N=1122)	MASH/F0 (N=369)	MASH/F1 (N=934)	MASH/F2 (N=382)	MASH/F3 (N=327)	*p-*trend
Cirrhosis or decompensated liver disease, N	486	117	38	121	78	132	
Person-years	20,604	7771	2502	6294	2501	1535	
Incidence rate^a^ per 1000 PY [95% CI]	23.59 [21.54–25.78]	15.06 [12.45, 18.04]	15.18 [10.74, 20.84]	19.22 [15.95, 22.97]	31.19 [24.65, 38.92]	85.98 [71.94, 101.96]	—
Absolute rate difference^b^, [95% CI]	—	—	0.13 [−5.42, 5.67]	4.17 [−0.21, 8.55]	16.13 [ 8.69, 23.57]	70.92 [56.00, 85.84]	—
Multivariable-adjusted HR^c^ [95% CI]	—	—	1.04 [0.72, 1.5] *p*=0.84	1.19 [0.92, 1.54] *p*=0.18	1.89 [1.41, 2.52] *p*<0.005	4.21 [3.26, 5.43] *p*<0.005	<0.005

a CIs for incidence rates and absolute rate differences were approximated by the normal distribution.

b Absolute risks and absolute risk differences [percentage points] were calculated based on Kaplan-Meier estimates.

c The multivariable-adjusted model accounted for age at the index date, sex, BMI, diabetes, dyslipidemia, smoking status, aspirin therapy, and statin therapy.

Abbreviations: BMI, body mass index; MASH, metabolic dysfunction–associated steatohepatitis; MASLD, metabolic dysfunction–associated steatotic liver disease; PY, person-years.

Because of the heterogeneous nature of the primary endpoint which included biopsy-proven NLP-detected cirrhosis, validated ICD9/10-coded cirrhosis, or evidence of decompensated cirrhosis, we also examined cirrhosis separately from decompensating events (Table 5). Examining the outcome of cirrhosis in isolation, the incidence rates of MASH/F0 were not significantly changed compared to the combined primary endpoint analysis, except steatosis which was more definitively less severe than MASH F0 and MASH F1. This did not reach statistical significance; however, in the sensitivity analysis further excluding events occurring within 30 days of index biopsy, MASH/F1 reached statistical significance for increased risk of cirrhosis as compared to steatosis (Supplemental Table S9, http://links.lww.com/HC9/A837).

**TABLE 5:** Longitudinal cohort risk analysis for secondary outcomes

	All MASLD (N=3134)	Simple steatosis (N=1122)	MASH/F0 (N=369)	MASH/F1 (N=934)	MASH/F2 (N=382)	MASH/F3 (N=327)	*p-*trend
Risk of cirrhosis							
Cirrhosis, N	343	39	33	87	63	121	
Person-years	21,082	8014	2532	6417	2534	1584	
Incidence rate^a^ per 1000 PY [95% CI]	16.27 [14.59–18.09]	4.87 [3.46, 6.65]	13.03 [8.97, 18.30]	13.56 [10.86, 16.72]	24.86 [19.10, 31.81]	76.41 [63.40, 91.30]	—
Absolute rate difference^b^, [95% CI]	—	—	8.17 [ 3.46, 12.87]	8.69 [ 5.46, 11.92]	19.99 [13.67, 26.32]	71.55 [57.85, 85.25]	—
Multivariable-adjusted HR^c^ [95% CI]	—	—	2.66 [1.66, 4.25] *p*<0.005	2.56 [1.75, 3.74] *p*<0.005	4.64 [3.1, 6.95] *p*<0.005	11.94 [8.27, 17.24] *p*<0.005	<0.005
Risk of decompensated cirrhosis event							
Decompensated cirrhosis event, N	267	93	19	60	35	60	
Person-years	20,604	7771	2502	6294	2501	1535	
Incidence rate^a^ per 1000 PY [95% CI]	12.18 [10.76, 13.73]	11.76 [9.49, 14.40]	7.18 [4.33, 11.22]	9.06 [6.92, 11.66]	12.60 [8.78, 17.53]	30.50 [23.28, 39.27]	—
Absolute rate difference^a^, [95% CI]	—	—	−4.57 [−8.59, −0.55]	−2.69 [−6.01, 0.62]	0.85 [−3.96, 5.66]	18.75 [10.67, 26.83]	—
Multivariable-adjusted HR^c^ [95% CI]	—	—	0.72 [0.43, 1.18] *p*=0.19	0.76 [0.55, 1.06] *p*=0.11	1.03 [0.69, 1.52] *p*=0.9	2.03 [1.46, 2.83] *p*<0.005	<0.005
Risk of HCC							
HCC, N	123	70	7	14	9	23	
Person-years	20,643	7679	2518	6350	2530	1566	
Incidence rate^a^ per 1000 PY [95% CI]	5.52 [4.59, 6.59]	8.95 [6.98, 11.30]	2.60 [1.04, 5.35]	2.07 [1.13, 3.47]	3.15 [1.44, 5.98]	10.78 [6.83, 16.18]	—
Absolute rate difference^b^, [95% CI]	—	—	−6.35 [−9.20, −3.51]	−6.88 [−9.24, −4.52]	−5.80 [−8.74, −2.86]	1.83 [−3.05, 6.71]	—
Multivariable-adjusted HR^c^ [95% CI]	—	—	0.30 [0.14, 0.66] *p*<0.005	0.22 [0.12, 0.4] *p*<0.005	0.33 [0.16, 0.66] *p*<0.005	0.94 [0.58, 1.54] *p*=0.82	0.34

a CIs for incidence rates and absolute rate differences were approximated by the normal distribution.

b Absolute risks and absolute risk differences [percentage points] were calculated based on Kaplan-Meier estimates.

c The multivariable-adjusted model accounted for age at the index date, sex, BMI, diabetes, dyslipidemia, smoking status, aspirin therapy, and statin therapy.

Abbreviations: BMI, body mass index; MASH, metabolic dysfunction–associated steatohepatitis; MASLD, metabolic dysfunction–associated steatotic liver disease; PY, person-years.

Finally, we considered biopsies with borderline MASH characteristics. These were biopsies with steatosis and ballooning without lobular inflammation (n=63) and biopsies with lobular inflammation without ballooning (n=240) that were excluded from the primary analysis. Multivariable risk analysis was not feasible to conduct on these categories separately due to the low sample size. As a sensitivity analysis, steatotic biopsies with only lobular inflammation were categorized as simple steatosis, while steatotic biopsies with only ballooning were categorized as MASH/F0 based on Kleiner et al^26^ (Supplemental Table S10, http://links.lww.com/HC9/A837). Including these additional cases did not change the significance or order of risk for any category, but did accentuate the trend toward the significance of MASH/F0 and MASH/F1 risk of advanced liver disease in comparison to simple steatosis.

### Risk of HCC

We also evaluated the risk of HCC as a secondary outcome using the same index biopsy exposure cohort as above (n=3134; 20,642 person-years). HCC was overall rare (Table 5), occurring 123 times using the gold standard ICD codes (Definitions of outcomes, Supplemental Materials, http://links.lww.com/HC9/A837). The highest incidence rate occurred among patients with an index biopsy demonstrating F3 fibrosis (10.78 events per 1000 person-years). However, the second highest category was steatosis, with both raw incidence rates and multivariable-adjusted hazard rates demonstrating reduced risk among patients with F0-F2 compared to simple steatosis.

In sensitivity analysis where HCC events occurring within 30 days of index biopsy were excluded, over half of the cases were eliminated (Supplemental Table S11, http://links.lww.com/HC9/A837, n=74). The absolute incidence of HCC given an index biopsy showing steatosis, MASH/F0, or MASH/F1 fell significantly compared to the baseline analysis. The multivariable HR for HCC rose steadily from MASH/F1 to MASH/F3, although the *p*-trend was not significant, and none of the categories were statistically significantly higher than simple steatosis.

## DISCUSSION

Histopathology remains the gold standard in the diagnosis and staging of human MASLD.^6^^,^^8^ Despite histopathology reports being widely available in center-level databases, these reports remain a largely untapped resource due to their free-text nature. This study establishes that an NLP algorithm pre-trained on medical corpuses,^24^ coupled with negation detection,^23^ can automatically detect pathologist-described liver histopathology with performance characteristics that rival human validators, the traditional gold standard. Deployment of NLP has the potential to save time, reduce errors, and enable large-scale analysis of pathology data that would be infeasible manually.

As proof of concept, we deployed the algorithm to automatically score liver biopsies across a diverse multicenter health care system, to create 2 large MASLD cohorts. The first cohort, which included nearly 9000 patients, provided a cross-sectional view of patients with MASLD at the time of their liver biopsy. Cohort characteristics matched known clinical phenomena in human steatosis and MASH, including steadily rising transaminases (ALT>AST) across the fibrosis stage before declining in the setting of cirrhosis, and PT-INR which becomes abnormal at the cirrhosis stage. These data also confirm a reduction in dyslipidemia at the onset of cirrhosis, as described.^29^^,^^30^

The second cohort examined rates of progression of MASLD to advanced liver disease among 3134 patients. To our knowledge, this is the largest study of disease progression stratified by fibrosis stage yet conducted and offers patients and clinicians a more precise prognosis based on index liver biopsy staging. We find an accelerating risk of progression to cirrhosis with increasing fibrosis stage, mirroring and validating the results of a meta-analysis (n=1495) which found a similar accelerating trend in mortality for patients with later fibrosis stages.^31^ Other studies examined fibrosis progression by pooling paired liver biopsy studies^32^ or analyzing the placebo arms of clinical trials,^33^ and estimated rates of 0.14 (n=116) or 0.03 (n=952) fibrosis stages per year, respectively. We find an incidence rate of 0.083 (0.072–0.10, 95% CI, Table 4) for single-stage advancement of F3 to F4, which is comparable to prior estimates (n=3134). As an exercise for comparison purposes, and assuming linear progression by standardizing our rates on the scale of a single F-stage yields 0.062 stages per year (F2), 0.058 stages per year (F1), and 0.061 stages per year (F0), a rate that does not significantly differ if an index biopsy shows steatosis without MASH (0.060 stages per year). Prior studies were not stratified by index fibrosis stage, but single-stage progression of patients with simple steatosis to F1 was previously estimated to be 0.05,^32^ similar to our estimate here.

The stratification of risk by fibrosis stage has significant implications for the study of human MASLD.^34^^,^^35^ The apparent disease acceleration at F3, mirrored by accelerated mortality rates,^31^ suggests clinical trials may benefit from focusing on patients with F3 fibrosis. Not only are patients with F3 at the greatest risk of progression to cirrhosis, but their accelerated risk indicates fewer patients would need to be followed, and for less time, to achieve meaningful endpoints. In contrast, our MASH/F0 absolute incidence rate of 15.18 per 1000 patient years corresponds to an average time-to-event of 66 years. Coupled with a mean age of biopsy for MASH/F0 being 47 years, patients with MASH/F0 are on average unlikely to progress to cirrhosis in their natural lifespan. Our analysis indicates the same may be true of both simple steatosis and even MASH/F1, and may explain the failure of many clinical trials to achieve significant endpoints. Our observation lends credibility to the theory of rapid progressors, a notion raised by paired biopsy studies,^32^^,^^36^^,^^37^ among which the largest study estimated ~14%–20% of patients had >1 fibrosis stage progression in short interval paired biopsies.^32^ While numerous biomarkers for MASH exist, additional scrutiny is needed to differentiate rapid progressors, especially among patients with early stages of the disease. Finally, if confirmed at other centers, our results may revise epidemiologic projections of MASH burden on liver transplantation.

There are some limitations to this study. Our biopsy report data set is intrinsically heterogeneous, covering more than 3 decades during which MASLD definitions shifted,^8^ and representing the assessments of several dozen pathologists evaluating biopsies according to routine clinical judgment. To address stylistic heterogeneity, the algorithm was necessarily reductive, for example simplifying histologic grades for steatosis, inflammation, and ballooning to a binary presence or absence even when the grade was reported. Incorporating histologic grading in future algorithm versions is attractive, and feasible, at the cost of introducing considerable missingness, especially to pre-2005 pathology reports. However, this also highlights a strength of the current study being a “real-world” test of MASH CRN grading and staging, with preservation of the key observation that fibrosis stage predicts cirrhosis risk.

In addition, our estimated single-stage rates may be lower than prior estimates because our primary outcome includes evidence of decompensated cirrhosis, whereas paired biopsy studies were more likely to capture evolution to compensated cirrhosis. This limitation is somewhat addressed in our sensitivity analysis by examining cirrhosis alone, which enriched for biopsy-confirmed cirrhosis and did not yield significantly altered incidence rates. Future analyses where our NLP algorithm is replicated at other centers may enable sufficient sample size to conduct the more definitive paired biopsy study analysis. Related, though we used validated ICD codes to define our outcomes, this is inherently less precise than those defined by biopsy or radiology. As an example of uncertainty intrinsic to ICD-code–based outcome ascertainment, our HCC cases were halved by excluding diagnoses within 30 days of biopsy, the majority of which derived from patients with a biopsy showing steatosis (Table 5, Supplemental Table S11, http://links.lww.com/HC9/A837). On chart review, many of these cases appear to be colorectal metastases, but for which the treating physician coded for HCC as the indication for liver biopsy. Our work offers extreme precision in the definition of our exposure (liver biopsy histologic status), but further attention is needed to more accurately identify clinical outcomes (HCC and decompensated cirrhosis). In addition, future studies should account for competing risks of these outcomes, like death or transplantation; as such, the risks reported here may be overestimated. Finally, this study encompasses multiple hospitals but within a single hospital system in the Northeast; future studies encompassing a more representative sampling of national geographic demographics may further refine our incidence rate estimates.

In conclusion, we introduce an NLP algorithm to score MASLD histopathology according to field-standard definitions. This automation proved reliable and helpful in the creation of high-quality MASLD cohorts and may be easily adapted to any free-text medical document. Our study identified an accelerating trend for developing cirrhosis by index fibrosis stage, suggesting that early-stage patients are on average unlikely to progress to cirrhosis in their natural lifespan. We anticipate our specific MASLD pathology NLP algorithm, and the overall methodology, will accelerate insight into this important and growing disease.

## Supplementary Material
