# Supplementary material for: A natural language processing algorithm accurately classifies steatotic liver disease pathology to estimate the risk of cirrhosis
Source: Hepatol Commun. 2024 Mar 29;8(4):e0403. doi: 10.1097/HC9.0000000000000403 (PMC10984665; doi:10.1097/HC9.0000000000000403)
Supplement: Supplementary file 1 [file hc9-8-e0403-s001.docx]

**Supplemental Materials**

**Natural language processing algorithm accurately classifies steatotic liver disease histopathology to estimate risk of cirrhosis and decompensation**

Marc S. Sherman MD, PhD^1,2,3^, Prasanna K. Challa^1^, Eric M. Przybyszewski MD^1^, Robert Wilechansky MD^1^, Eugenia Uche-Anya MD^1^, Ashley T. Ott MD^4^, Jessica McGoldrick^1^, Wolfram Goessling MD, PhD^1,2,3^, Hamed Khalili MD^1,3^, Tracey G. Simon MD^1,3^.

**MASLD Cohort Exclusion Criteria**

Exclusion criteria were (1) presence of any validated ICD codes for alcohol-related disease^26^ (ICD9: 357.5, 425.5, 535.3, 980.1, 980.9, 571.0, 571.1, 571.2, 571.3, 535.3, 305.0, 291, 303, T51.0, T51.9; ICD10: E24.4, G62.1, I42.6, K29.20, G31.2, G72.1, K86.0, T51.0, T51.9, Y57.3, X65, Z50.2, Z71.4, Z72.1, F10, K70, K85.2, K29.2, Z71.4). In addition, a small number of alcohol use exclusions were also made based on internal codes (LMR: LPA13, LPA12, LPA1535; Oncall: YHCJ1, YHAD5, QJPX3), DRG codes (202, 521, 433), APDRG codes (280, 770, 775), and DSM-IV codes (305.0, 303.9.1). (2) positive mention (as defined by NLP output) of a history of alcohol (“alcoholic”, “ethanol”) in the biopsy report, (3) NLP-defined assessment of the presence of any other liver disease in the biopsy report. (4) Post-transplantation and explants as denoted by the terms “allograft”, “donor”, “explant” and “native” within the report heading text. And finally, (5) patients with medication administration history of prednisone, amiodarone, and methotrexate.

**Definitions of comorbidities**

Diabetes was defined by an ICD-9 code for V58.67, 250.0, 250.00, 250.02, 250.1, 250.10, 250.12, 250.4, 250.40, 250.42, 250.5, 250.50, 250.52, 250.6, 250.60, 250.62, 250.7, or 250.70 or an ICD-10 code starting with E11*. Dyslipidemia was defined by an ICD9 code for ICD9: 272.0, 272.1, 272.2, 272.3, 272.4, 272.5 or an ICD-10 code for E78.0, E78.1, E78.2, E78.3, E78.4, or E78.5. Hypertension was defined by an ICD-9 code starting with any of 401.*, 402.*, 403.*, 404.*, or 405 or an ICD-10 code starting with I10.*, I11.*, I12.*, I13.*, I14.*, or I15.*. Statin use and aspirin use were obtained from MGB-system medication prescription documentation. Smoking status was extracted from physician documentation of smoking status in clinical documentation. Bariatric surgery was defined by ICD9 codes (ICD9: 43.82, 43.89, 44.31, 44.38, 44.39, 44.68, 44.95)^19^, CPT codes (43644, 43645, 43770, 43771, 43772, 43773, 43775, 43842, 43843, 43846, 43847, 43848, 43845)^19^, with some refinement of the published lists to exclude bariatric device removal codes. In addition, HCPCS codes (S2082, S2085)^20^ and DRG codes (288, 619, 620, 621)^20^ were also used to identify bariatric surgery cases. Heights, weights and BMI were obtained from a documented vitals database.

**Definitions of outcomes**

(1) Cirrhosis was identified by ICD codes (ICD-9: 571.5; ICD-10: K74.6)^21^ or a subsequent NLP-detected biopsy report indicating cirrhosis. (2) Decompensated liver disease events was defined as a composite including any of ascites (ICD-9: 789.5; ICD-10: R18)^21^, spontaneous bacterial peritonitis (SBP) (ICD-9: 567.23; ICD-10: K65.2)^21^, varices (ICD-9: 456.1, 456.21, 456.0, 456.20; ICD-10: I85.9, I98.2, I86.4, I85.0, I98.3)^21^, hepatorenal syndrome (HRS) (ICD9: 572.4; ICD10: K76.7)^21^, or hepatic encephalopathy (HE) (ICD-9: 572.2). The primary outcome was considered cirrhosis or a decompensated liver event, either (1) or (2). (3) Hepatocellular carcinoma (HCC) was defined by ICD codes (ICD-9 155.0, 155.2; ICD-10: C22.0, C22.9)^21^.

**Tables**

**Table S1.** **Performance characteristics of NLP algorithm for prediction of MASLD concepts – Massachusetts General Hospital (517 reports)**

| Concept | Sensitivity/  Recall % | Specificity % | PPV/  Precision% | NPV % | F1 Score | Support |
| --- | --- | --- | --- | --- | --- | --- |
| “steatosis” | 100.0 | n/a | 99.8 | nan | 99.9 | 516 |
| “lobular inflammation” | 92.1 | 99.7 | 99.5 | 95.2 | 95.6 | 202 |
| “ballooning degeneration” | 95.5 | 100.0 | 100.0 | 97.2 | 97.7 | 202 |
| “NASH” | 98.8 | 97.3 | 97.3 | 98.8 | 98.1 | 255 |
| “cirrhosis” | 97.9 | 99.0 | 95.9 | 99.5 | 96.9 | 96 |
| Brunt/Fibrosis F0 | 95.8 | 99.0 | 96.6 | 98.8 | 96.2 | 118 |
| Brunt/Fibrosis F1 | 93.6 | 98.8 | 95.3 | 98.3 | 94.4 | 109 |
| Brunt/Fibrosis F2 | 96.9 | 98.6 | 94.1 | 99.3 | 95.5 | 98 |
| Brunt/Fibrosis F3 | 98.0 | 99.5 | 98.0 | 99.5 | 98.0 | 99 |
| Brunt/Fibrosis F4 | 96.7 | 98.8 | 94.6 | 99.3 | 95.7 | 91 |

**Table S2.** **Performance characteristics of NLP algorithm for prediction of MASLD concepts – Brigham and Women’s (409 reports)**

| Concept | Sensitivity/  Recall % | Specificity % | PPV/  Precision% | NPV % | F1 Score | Support |
| --- | --- | --- | --- | --- | --- | --- |
| “steatosis” | 100 | n/a | 99.5 | n/a | 99.8 | 407 |
| “lobular inflammation” | 89.0 | 99.4 | 97.3 | 97.3 | 92.3 | 82 |
| “ballooning degeneration” | 96.4 | 100.0 | 100.0 | 99.4 | 98.2 | 56 |
| “NASH” | 97.8 | 98.2 | 96.3 | 98.9 | 97.0 | 134 |
| “cirrhosis” | 98.9 | 99.7 | 98.9 | 99.7 | 98.9 | 91 |
| Brunt/Fibrosis F0 | 96.7 | 99.7 | 98.9 | 99.1 | 97.8 | 92 |
| Brunt/Fibrosis F1 | 95.0 | 99.4 | 97.4 | 98.8 | 96.2 | 80 |
| Brunt/Fibrosis F2 | 97.4 | 98.5 | 93.8 | 99.4 | 95.6 | 78 |
| Brunt/Fibrosis F3 | 98.5 | 99.4 | 97.1 | 99.7 | 97.8 | 68 |
| Brunt/Fibrosis F4 | 98.9 | 99.7 | 98.9 | 99.7 | 98.9 | 91 |

**Table S3.** **Performance characteristics of NLP algorithm for prediction of MASLD concepts – Other hospitals (98 reports)**

| Concept | Sensitivity/  Recall % | Specificity % | PPV/  Precision% | NPV % | F1 Score | Support |
| --- | --- | --- | --- | --- | --- | --- |
| “steatosis” | 100 | n/a | 98.98 | n/a | 99.5 | 97 |
| “lobular inflammation” | 90.9 | 100.0 | 100.0 | 93.1 | 95.2 | 44 |
| “ballooning degeneration” | 100.0 | 100.0 | 100.0 | 100.0 | 100.0 | 46 |
| “NASH” | 100.0 | 97.7 | 98.2 | 100.0 | 99.1 | 55 |
| “cirrhosis” | 100.0 | 100.0 | 100.0 | 100.0 | 100.0 | 16 |
| Brunt/Fibrosis F0 | 92.9 | 98.8 | 92.9 | 98.8 | 92.3 | 13 |
| Brunt/Fibrosis F1 | 88.9 | 98.6 | 96.0 | 95.9 | 92.3 | 27 |
| Brunt/Fibrosis F2 | 100.0 | 97.5 | 89.5 | 100.0 | 94.4 | 17 |
| Brunt/Fibrosis F3 | 100.0 | 100.0 | 100.0 | 100.0 | 100.0 | 24 |
| Brunt/Fibrosis F4 | 100.0 | 100.0 | 100.0 | 100.0 | 100.0 | 16 |

**Table S4.** **Performance characteristics of NLP algorithm for negative prediction of steatosis – All sites (100 MGH reports, 100 non MGH reports)**

| Concept | n | Sensitivity/  Recall % | Specificity % | PPV/  Precision% | NPV % | F1 Score | Support |
| --- | --- | --- | --- | --- | --- | --- | --- |
| “steatosis” | 200 | 0.0 | 100.0 | nan | 99.5 | nan | 1 |

**Table S5. Arbitrating algorithm-validator disagreement (combined).**

|  | | |  |  |  |  |  |
| --- | --- | --- | --- | --- | --- | --- | --- |
|  | Steatosis | Inflammation | Ballooning | Cirrhosis | MASH | Fibrosis stage | Total (%) |
| Disagreements, n | 7 | 49 | 15 | 10 | 35 | 60 | 176 |
| Algorithm correct, n | 3 | 17 | 4 | 2 | 16 | 22 | 64 (36) |
| Validator correct, n | 4 | 32 | 11 | 8 | 19 | 35 | 109 (62) |
| 3-way disagreement, n | - | - | - | - | - | 3 | 3 (2) |

**Table S6. Arbitrating algorithm-validator disagreement (separated by sites)**

| **MGH** | | | | | | | |
| --- | --- | --- | --- | --- | --- | --- | --- |
|  | Steatosis | Inflammation | Ballooning | Cirrhosis | MASH | Fibrosis stage | Total (%) |
| Disagreements, n | 3 | 21 | 10 | 6 | 15 | 25 | 80 |
| Algorithm correct, n | 2 | 5 | 1 | 0 | 5 | 3 | 16 (20) |
| Validator correct, n | 1 | 16 | 9 | 6 | 10 | 20 | 62 (77) |
| 3-way disagreement, n | 0 | 0 | 0 | 0 | 0 | 2 | 2 (3) |
| **BWH/other** | | | | | | | |
| Disagreements, n | 4 | 28 | 5 | 4 | 20 | 35 | 96 |
| Algorithm correct, n | 1 | 12 | 3 | 2 | 11 | 19 | 48 (50) |
| Validator correct, n | 3 | 16 | 2 | 2 | 9 | 15 | 47 (49) |
| 3-way disagreement, n | 0 | 0 | 0 | 0 | 0 | 1 | 1 (1) |

**Table S7.** **Risk of advanced liver disease excluding outcomes occurring within 30 days of inclusion (sensitivity analysis for Table 4)**

**Table S8. Risk of advanced liver disease outcome excluding patients with missing BMI (sensitivity analysis for Table 4)**

**Table S9. Risk of cirrhosis outcome excluding outcomes occurring within 30 days of inclusion (sensitivity analysis for Table 5)**

**Table S10. Risk of advanced liver disease including borderline MASH categories (sensitivity analysis for Table 4).**

**Table S11. Risk of HCC outcome excluding HCC diagnoses within 30 days of inclusion (sensitivity analysis for Table 5, HCC).**

**Table S12. Risk of decompensated cirrhosis event (sensitivity analysis to Table 5 excluding events occurring within 30 days of biopsy).**
